# Synergistic Effects of Interferon-γ and Vitamin D_3_ Signaling in Induction of ILT-3^high^PDL-1^high^ Tolerogenic Dendritic Cells

**DOI:** 10.3389/fimmu.2019.02627

**Published:** 2019-11-13

**Authors:** Urban Švajger, Primož J. Rožman

**Affiliations:** ^1^Blood Transfusion Center of Slovenia, Ljubljana, Slovenia; ^2^Faculty of Pharmacy, University of Ljubljana, Ljubljana, Slovenia

**Keywords:** vitamin D_3_, interferon-γ, tolerogenic DCs, dendritic cell, regulatory, PDL1, ILT-3, immunosuppression

## Abstract

In the past, interferon (IFN)-γ and vitamin D_3_ (vit D_3_) have both been associated with induction of tolerogenic characteristics in human dendritic cells (DCs). Although there are only a few reports on interdependency of their actions, the interplay between IFN-γ and vit D_3_ has been clearly demonstrated in certain aspects of immune reactivity. Since both agents have been associated with regulation of immune responses, we set out to examine their functional and mechanistic interactions in context of principal regulators of immunity, the DCs. Combined treatment with vit D_3_ and IFN-γ caused an extensive expression of immunoglobulin-like transcript (ILT)-3 and programmed death ligand (PDL)-1 on γ/D_3_DCs, significantly greater than that caused by vit D_3_ alone. Such γ/D_3_DCs retained all general DC characteristics. After CD40 ligand-induced activation, they produced increased amounts of IL-10 with almost absent production of IL-12p70. On the other hand, the co-stimulatory potential of γ/D_3_DCs was weak, with cells possessing the capacity to inhibit CD4^+^ T cell, CD8^+^ T cell, as well as memory T cell responses. Naive CD4^+^ T cells stimulated with γ/D_3_DCs produced increased amounts of IL-10 with concomitantly low IFN-γ production, upon T cell receptor activation. Additionally, γ/D_3_DCs completely inhibited granzyme B expression by CD8^+^ T cells. The percentage of FoxP3-positive cells in co-cultures with naive CD4^+^ T cells was significantly higher where γ/D_3_DCs were used as stimulators compared to DCs treated with vit D_3_ alone and it could be partially reversed by PDL-1 blockade. Interestingly, γ/D_3_DCs were inefficient at suppressing mDC-induced CD4^+^ T cell proliferation, but were twice as effective as D_3_DCs at suppressing mDC-induced CD8^+^ T cell proliferation. Blockade of indoleamine-2,3-dioxygenase did not reduce the tolerogenic phenotype induced by IFN-γ and vit D_3_ treatment. Examination of signaling pathways activation revealed a tendency toward increased ERK and Akt phosphorylation in γ/D_3_DCs. Inhibition of MEK/ERK and PI3K/mTOR pathways significantly reduced the expression of ILT-3 and PDL-1 on γ/D_3_DCs. In summary, we present the first evidence for existing synergy between IFN-γ and vit D_3_ in shaping a unique tolerogenic DC activation state.

## Introduction

The active metabolite of vitamin (vit) D (1,25-dihidroxycholecalciferol or vit D_3_) and interferon (IFN)-γ are both pleiotropic endogenous immune modulators and can assist in immunogenic and tolerogenic processes. The physiological functions of vit D_3_ extend well-beyond its recognized role in calcium homeostasis and almost every immune cell type carries vit D receptors (VDRs) ranging from T cells, B cells, neutrophils, and antigen (Ag)-presenting cells (APCs) (1, 2). The immunomodulatory effect of vit D_3_ on these cell types is considerable and can affect immune cell chemotaxis, activation, antibody production, differentiation, and APC maturation. Very often, vit D_3_-exerted effects on immune cells are associated with immunological tolerance induction, leading to induction of dendritic cells (DCs) with tolerogenic properties and skewing of T cell responses toward Th2-type effectors and regulatory T cells (Tregs) (3–5). On the other side, the presence vit D_3_ has been found crucial for optimal activation of monocytes/macrophages (6, 7).

Interferon-γ is a widely recognized pro-inflammatory cytokine produced by natural killer cells, Th1 cells, cytotoxic T cells, DCs, as well as innate lymphoid cells (8–10). However, the role of IFN-γ in orchestrating a tolerogenic immunological outcome has also been demonstrated on numerous occasions both *in vitro* and *in vivo* (11). For example, IFN-γ is an important inducer of indoleamine-2,3-dioxygenase (IDO), an immunosuppressive enzyme involved in tryptophan catabolism (12). Additionally, we have shown previously that when DCs are treated with high doses of IFN-γ in the absence of danger signals, they obtain extensive tolerogenic characteristics and are capable of suppressing cyototoxic T cell responses in a HLA-G-dependent manner (13).

The early observations that this hormone and cytokine could be engaged in mutually-dependent regulation of immune reactivity date back more than 30 years. At that time Rook et al. demonstrated the increased anti-tuberculosis activity of human monocytes when treated with vit D_3_ in addition to IFN-γ stimulation (14, 15). The IFN-γ-mediated activity of monocytes/macrophages in terms of autophagy, antimicrobial peptide expression, and phagosome-lysosome fusion is today known to be strongly dependent on adequate presence of vit D_3_ (16). Pathway interactions between IFN-γ and vit D_3_ already appear at transcription level. As shown by Vidal et al., IFN-γ treatment induces increased nuclear translocation of the vit D receptor (VDR) (17). In this manner, increased nuclear presence of both VDR and STAT-1 leads to functional STAT-1/VDR interactions, which in turn enhances IFN-γ signaling by preventing STAT-1 dephosphorylation. In disease setting, the ameliorating effect of vit D_3_ on experimental autoimmune encephalomyelitis (EAE) development was shown to be 2-fold less prominent in *ifng* gene knockout mice (18). The expression of IFN-γ was thus needed for *vdr* gene expression and vit D_3_-dependent mechanisms which inhibited EAE development. The functional outcomes of interactions between IFN-γ and vit D_3_ therefore also seem to differ in terms of immune reactivity, suggesting dependence on pathology in question and immune cell types involved.

Dendritic cells represent the most important APCs, linking innate and adaptive immune responses, with the capacity of controlling both immunity and tolerance (19). Vitamin D_3_ can induce potent tolerogenic characteristics in DCs by strongly up-regulating various surface inhibitory molecules and increasing their capacity to produce IL-10 upon activation (20). While IFN-γ is well-known for acting as a priming agent in type 1 DC activation, leading to enhanced co-stimulation and IL-12 production, the outcome of its signaling is greatly dependent on the presence of concurrent signaling from other factors in DC microenvironment. It is thus clear that both IFN-γ and vit D_3_ have important functional roles in the regulation of DC biology. Due to the fact that pathways of both endogenous immune modulators have been shown to interact in other cell types at least to a certain extent, we set out to explore their potential mutual contribution in regulating the fundamental aspects of DC function.

## Materials and Methods

This section has been completed according to MITAP (Minimum Information for Tolerogenic Antigen Presenting cells) guidelines (21).

### Cell Isolation and Culture

Buffy coats (~55 ml) from venous blood of normal healthy volunteers (*homo sapiens*) were obtained by the Blood Transfusion Centre of Slovenia, according to institutional guidelines and approval of the National Medical Ethics committee of Slovenia under number 0120-279/2017-3 and used within 6 h. Peripheral blood mononuclear cells (PBMCs) were isolated using Lympholyte^®^-H (Cedarlane laboratories, Ontario, Canada). The cells were washed twice with Dulbecco's phosphate-buffered saline (DPBS), counted, and used as a source for immunomagnetic isolation of CD14-positive cells (Miltenyi Biotec GmbH, Bergisch Gladbach, Germany). The purity of monocytes was always >90%, determined by flow cytometry. These were cultured in RPMI 1640 (Cambrex) medium supplemented with 10% fetal bovine serum (FBS), Gentamicin (50 g/ml; Gibco, Paisley, UK), 800 U/ml of rhGM-CSF, and 1000 U/ml of rhIL-4 (both Peprotech EC, London, UK), at 37°C, 5% CO_2_. The concentration of monocytes during differentiation was 0.5 × 10^6^ cells/ml. On day 2, half of the medium was exchanged with starting quantities of rhGMCSF (800 U/ml) and rhIL-4 (1000 U/ml). After 5–6 days non-adherent iDCs were harvested and characterized by flow cytometry as CD1a^hi^, CD14^−^, DC-SIGN^hi^, CD80^low^, CD83^−^, CD86^low^, and HLA-DR^low^. After microscopic examination, the DCs exhibited typical morphological characteristics of monocyte-derived DCs.

After differentiation, DCs were stimulated with 500 U/ml of IFN-γ (Peprotech, London, UK) or various concentrations of 1,25-dihidroxycholecalciferol (vit D_3_) (0.1–10 ng/ml) (Sigma Aldrich) for 24 h. For controls we used either unstimulated, immature DCs (iDCs) or mature DCs (mDCs) activated with lipopolysaccharide (LPS, 20 ng/ml) (Sigma Aldrich) and IFN-γ (500 U/ml). In certain experiments, 25-hidroxycholecalciferol (25(OH)D) was used at a concentration of 50 ng/ml. The terminology of variously treated DCs is determined as γDCs (IFN-γ-treated), D_3_DCs (vit D_3_ treated), or γ/D_3_DCs (simultaneously treated with IFN-γ and vit D_3_). In some cases, the induction of tolerogenic DCs was followed by 24 h exposure to LPS (20 ng/ml) or CD40 ligand multimer kit (mCD40L) (2 μg/ml of recombinant CD40L) (Miltenyi Biotec GmbH, Bergisch Gladbach, Germany). The induction of DC tolerogenic state or DC maturation/activation was performed in 48- or 24-well tissue culture plates with cell concentration 0.5 × 10^6^ cells/ml (0.5 × 10^6^ DCs and 1 × 10^6^ DCs in total for 48-well and 24-well culture plates, respectively). The DCs were always used fresh and never frozen.

T cells were purified from PBMCs. Whole CD4^+^ T cells were obtained by positive selection using CD4 microbeads (Miltenyi Biotec, GmbH). The purity of CD4^+^ cells was always >95% as determined by flow cytometry. Naïve CD4^+^CD45RA^+^ T cells were isolated using the naïve CD4^+^ T-cell isolation kit from Miltenyi Biotec, strictly following the manufacturer's protocol. The purity of isolated naïve CD4^+^ T cells was always >98%. Memory CD4^+^CD45RO^+^ T cells were isolated in a two-step procedure by first isolating untouched, whole CD4^+^ T cells by negative selection using CD4^+^ T cell isolation kit II (Miltenyi Biotec, GmbH) followed by positive selection of memory cells using CD45RO microbeads (Miltenyi Biotec, GmbH). Whole CD8^+^ T cells were obtained by positive selection using CD8 microbeads (Miltenyi Biotec, GmbH).

### Phenotypic Characterization and Endocytosis Studies

For flow cytometry analysis of DC phenotype we used the following monoclonal antibodies (mAbs): FITC-labeled anti-CD1a (clone: HI149), anti-CD1c (clone: L161), anti-CD14 (clone: 63D3), anti-CD16 (clone: 3G8), and anti-HLA I (clone: W6/32) (all from Biolegend, CA, USA); Alexa Fluor 488-labeled anti-CD4 (clone: RPA-T4) (Biolegend); PE-labeled anti-CD11b (clone: ICRF44), anti-CD11c (clone: 3.9), anti-DC-SIGN (clone: 9E9A8), anti-CD80 (clone: 2D10), anti-CD83 (clone: HB15e), anti-CD86 (clone: BU63) (all from Biolegend), and anti-HLA II (clone: AC122), anti-ILT-3 (clone: REA141), anti-ILT4 (clone: REA184), anti-PDL-1 (clone: REA1197), and anti-FasL (clone: NOK-1) (all from Miltenyi Biotec). Briefly, variously treated DCs were harvested and collected by centrifugation. Antibody was added and the cells were incubated for 15 min in the dark, then washed twice and resuspended in 2% paraformaldehyde (PFA). Samples were analyzed on a FACSCalibur system (Becton Dickinson, Inc.). Data was analyzed with the CellQuest software (BD biosciences).

Endocytosis was monitored by flow cytometry after incubation of DCs with FITC-dextran (1 mg/ml), either on ice or at room temperature, for 1 h. The cells were then washed twice with DPBS and re-suspended in 2% PFA for future analysis.

### Apoptosis Experiments

We used human peripheral-blood monocytes for differentiation of iDCs using GM-CSF (800 U/ml) and IL-4 (1000 U/ml). Dendritic cells were either left untreated or treated with IFN-γ (500 U/ml), vit D_3_ (10 ng/ml), or IFN-γ + vit D_3_, for 72 h. Viability of cells, as well as the percentage of early and late apoptotic cells was measured by Annexin V-FITC and 7-aminoactinomycin D (7-AAD) staining. Measurements were performed on a FACSCalibur flow cytometer (Beckton Dickinson).

### Morphological Cell Analysis

To analyze the cellular morphology of variously treated DCs we performed microscopy analysis using the inverted light optical microscope (Nikon Eclipse Ti-S, Tokyo, Japan).

### Quantification of Cytokine Production

The BD Human Inflammatory Cytometric Bead Array Kit (BD Biosciences) was used to assay the protein levels of IL-1β, IL-6, IL-8, IL-10, IL-12p70, and TNF-α in the cell culture supernatant, according to the manufacturer's protocol. In brief, the immature DCs collected on day 6 were washed twice with DPBS. Afterwards, the cells were either left unstimulated or stimulated with IFN-γ, vit D_3_ (10 ng/ml) or IFN-γ + vit D_3_. After 24 h, the cells were washed extensively and seeded in 48 wells (0.5 × 10^6^ cells/well) and stimulated with human mCD40L at 4 μg/ml (Miltenyi Biotec) for 24 h. At the end of cell culture the supernatants were harvested and used for further analysis. In an appropriate assay tube, 50 μl of the Capture Beads, 50 μl of the Detection Reagent and 50 μl of sample (5- or 10-fold dilution) were mixed and incubated for 3 h at room temperature and protected from light. Samples were then washed with 1 ml of Wash Buffer and centrifuged at 300 g for 5 min. The supernatant was carefully aspirated and discarded from each assay tube. The bead pellet was resuspended by adding 300 μl of Wash Buffer. Flow cytometry was performed using a FACSCalibur system (Becton Dickinson, Inc.). A standard curve was prepared by serial dilutions of standards and used for determination of cytokine concentrations in supernatants.

T cell activation and polarization was determined using the Human Th1/Th2/Th17 CBA kit (BD Biosciences). T cell polarization was determined after co-cultures of variously treated DCs with naive CD4^+^CD45RA^+^ T cells in RPMI 1640 + 10% human AB serum. Briefly, iDCs, mDCs, γDCs, D_3_DCs, or γ/D_3_DCs were co-cultured with naive T cells at 1:10 ratio in 48 wells (1 × 10^5^ DCs and 1 × 10^6^ T cells/well) for 7–9 days. At the end of co-culture, the T cells were collected, washed extensively, plated and re-stimulated with anti-CD2/CD3/CD28 macrobeads (T cell activation/expansion kit, Miltenyi Biotec) for 24 h. After re-stimulation, the T cell supernatants were collected and analyzed for the presence of IL-2, IL-4, IL-6, IL-10, IL-17A, IFN-γ, and TNF-α.

The activation of CD8 T cells was determined by analyzing the production of IFN-γ using the CBA assay. Variously treated DCs were co-cultured with whole CD8^+^ T cells in 48 well plates at 1:10 cell ratio in RPMI1640 + 10% human AB serum. After 5 days, the supernatants were collected and analyzed for the presence of IFN-γ.

### Allogeneic T Cell Proliferation

Variously treated DCs were assessed for their allo-stimulatory capacity. After differentiation, the DCs were either left in their immature state or treated with IFN-γ (500 U/ml), vit D_3_ (10 ng/ml), or combination of both. Mature DCs activated with IFN-γ (500 U/ml) + LPS (20 ng/ml) were used as positive controls. Briefly, variously treated DCs were used as stimulators in a mixed lymphocyte culture with either whole CD4^+^ T cells, whole CD8^+^ T cells or memory CD4^+^CD45RO^+^ T cells. In case of whole CD4^+^ T cells, neutralizing LEAF (low endotoxin, azide-free) anti-PDL-1 (clone: MIH2), anti-IL-10R (clone: 3F9), anti-FasL (clone: NOK-1), and anti-HLA-G (clone: 87G) mAbs (all from Biolegend) were used during co-cultures in concentration of 10 μg/ml. The co-cultures were performed in flat-bottom 96 wells, with each well-containing 2 × 10^4^ DCs and 2 × 10^5^ T cells in 300 μl of RPMI 1640 supplemented with 10% human AB serum. After 4 days, the wells were pulsed with 1 μCi/well ^3^H-thymidine (Perkin Elmer, Boston, MA) and proliferation measured by its incorporation after 18 h by liquid scintillation counting.

### DC Suppression Assay

Variously treated DCs were tested for their capacity to suppress T cell proliferation induced by fully mature DCs. The co-cultures were performed in flat-bottom 96 well, with each well containing mDCs as stimulators (2 × 10^4^ cells) and responding CD4^+^ or CD8^+^ T cells (2 × 10^5^) in 300 μl of RPMI1640 supplemented with 10% human AB serum. In some co-cultures we additionally added either iDCs, γDCs, D_3_DCs, or γ/D_3_DCs (2 × 10^4^). After 4 days, the wells were pulsed with 1 μCi/well ^3^H-thymidine (Perkin Elmer, Boston, MA) and proliferation measured by its incorporation after 18 h by liquid scintillation counting.

### Intracellular Staining

#### Phosflow Staining

Intracellular detection of signaling proteins pERK1/2, pAKT, and pSTAT1 was performed as described previously. Briefly, 1 × 10^5^ DCs were variously treated. After 60 min or 24 h, the cells were collected after fixation with addition of PFA directly into warm culture medium to ensure rapid freezing of signaling events. Afterward, cells were washed with PBS and permeabilized with ice-cold methanol for 10 min. Cells were then washed twice in PBS, and a solution of 3% BSA in PBS was added to prevent non-specific staining. Various signaling proteins were stained for their phosphorylation states with the following antibodies: PE anti-ERK1/2 (pT202/pY204) (clone: 20A), PE anti-pSTAT1 (pY701) (clone: K51-856) (both from BD biosciences, and PE anti-pAKT (pS473) (clone: REA134) (Miltenyi Biotec). Results were analyzed on a FACSCalibur system (Beckton Dickinson).

### Foxp3 Staining

Variously treated DCs were co-cultured with naive CD4^+^CD45RA^+^ T cells in RPMI1640 + 10% human AB serum. After 7 days, the T cells were collected and washed twice with DPBS. They were then rested for 24–48 h in RPMI 1640 + 10% AB with 10 U/ml of IL-2. The cells were then fixed using 2% PFA for 30 min. Afterwards, the cells were washed and permeabilized with FoxP3 staining Perm buffer (Biolegend, CA, USA). Levels of FoxP3 were then determined by staining for 45 min using PE-labeled, anti-FoxP3 (clone: 3G3) (Miltenyi Biotec). In some experiments, neutralizing LEAF anti-PDL-1 (Biolegend) was added in DC-T cell co-cultures at 10 μg/ml.

### Chemical Inhibitors Assays

Chemical inhibitors were used to assess involvement of various molecular mechanisms in DC tolerogenic characteristics. The inhibition of indoleamine-2,3-dioxygenase (IDO) was achieved using 10 μM of Epacadostat (Sigma Aldrich). We also used inhibitors of phosphoinositide-3 kinase (Ly294002 at 25 μM), MEK/ERK pathway (PD0325901, 1 μM), mTOR (Rapamycin at 100 nM), and STAT1 (Fludarabine at 50 μM). In all cases, DCs were pre-treated with inhibitors for 2 h. Afterwards, the DCs were treated with IFN-γ, vit D_3_ and IFN-γ + vit D_3_ for an additional 24 h. The DCs were then stained for the surface expression of ILT-3 and PDL-1, as described above.

### Statistical Analysis

Graphpad Prism software (v 6.07) was used for statistical analysis. Student's unpaired *t*-test was used for single comparisons, to detect statistical significance between individual pairs. *p* < 0.05 was considered statistically significant.

## Results

### γ/D_3_DCs Possess General DC Characteristics

**Figure 1:**
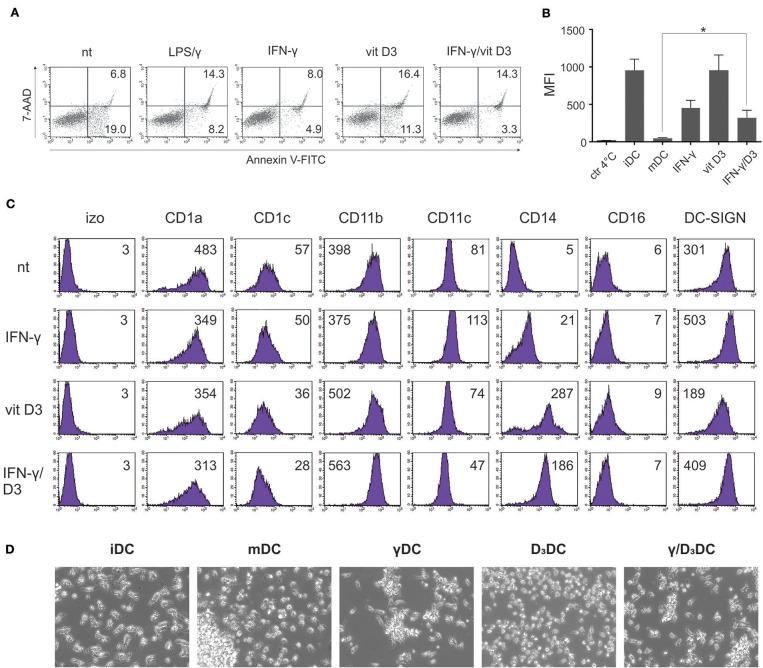
General characteristics of γ/D_3_DCs. Dendritic cells were fully differentiated from peripheral blood monocytes as described in section Materials and Methods. **(A)** Afterwards, the DCs were either left untreated or were stimulated with IFN-γ (500 U/ml), vit D_3_ (10 ng/ml), or combination of both. Mature DCs were obtained by stimulation with LPS (20 ng/ml) and IFN-γ (500 U/ml). After 72 h, the cells were harvested and the percentage of early apoptotic and dead cells determined by Annexin V-FITC and 7-AAD staining. The numbers in quadrants represent percentage of positive cells. Shown is one representative out of three independent experiments performed. **(B)** Variously treated DCs were incubated with FITC-labeled dextran for 1 h at 37°C, 5 %CO_2_. Afterwards the cells were washed twice and endocytosed FITC-dextran was determined by flow cytometry analysis. Incubation of cells at 4°C was used as a negative control. Shown is mean ± SD of four independent experiments. **(C)** Variously-treated DCs were stained for expression of broadly selected surface differentiation markers, as depicted in the figure, as described in section Materials and Methods. Shown is one representative out of four independent experiments performed. The numbers on histograms represent the mean fluorescence intensity (MFI) values. **(D)** Morphology of variously stimulated DCs was analyzed by inverted light microscopy. Shown is one representative out of three independent experiments performed. Statistical significance between individual pairs was calculated using Student's *t*-test. A *p*-value of less than 0.05 was considered statistically significant (^*^p < 0.05).

**Figure 2:**
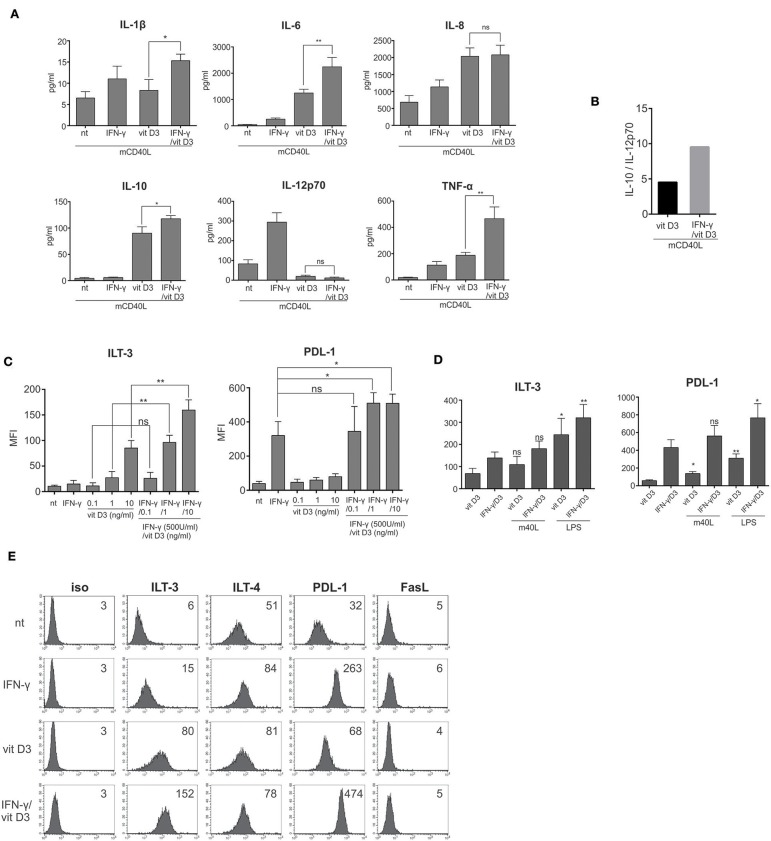
γ/D_3_DCs exhibit an exceptional tolerogenic phenotype. **(A)** Immature DCs were left untreated or treated with IFN-γ (500 U/ml), vit D_3_ (10 ng/ml), or IFN-γ + vit D_3_ at same concentrations. After 24 h, the DCs were washed extensively and re-stimulated using multiple CD40 ligand for an additional 24 h. Afterwards, the cell supernatants were analyzed for the presence of IL-1β, IL-6, IL-8, IL-10, IL-12p70, and TNF-α. Shown are mean ± SD of four independent experiments. **(B)** The ratio between the capacity of D_3_DCs and γ/D_3_DCs to produce IL-10 vs. IL-12p70 was calculated by dividing mean values of both cytokines, determined in cell culture supernatants after 24 h stimulation with CD40 ligand. **(C)** Immature DCs were treated with various concentrations of vit D_3_ (0.1 ng/ml, 1.0 ng/ml, or 10.0 ng/ml) in the presence or absence of 500 U/ml of IFN-γ. After 24 h, surface expression of ILT-3 and PDL-1 was determined using flow cytometry. Shown are mean ± SD of MFI values calculated on the basis of five independent experiments. **(D)** Dendritic cells were treated with vit D_3_ or IFN-γ + vit D_3_ for 24 h, followed by an addition 24 h exposure to LPS (20 ng/ml). Subsequently, expression of surface molecules ILT-3 and PDL-1 was analyzed and individual samples were compared to their counterpart, which was not stimulated with LPS. Shown are mean ± SD of MFIs of three independent experiments. **(E)** Dendritic cells were treated IFN-γ, vit D_3_, or combination of both and stained after 24 h for surface expression of different inhibitory molecules. Shown is one representative out of five independent experiments performed. The numbers on histograms represent MFI values. Statistical significance between individual pairs was calculated using Student's unpaired *t*-test (ns, non-significant; ^*^p < 0.05; ^**^p < 0.01).

We first set out to study the effects of simultaneous IFN-γ and vit D_3_ signaling on basic DC characteristics, such as cell viability, differentiation phenotype, endocytotic capacity, and general morphology. Dendritic cells were generated from human peripheral blood monocytes as described in section Materials and Methods. On day 6, CD1a^high^, CD14^neg^, DC-SIGN^high^ cells were either left untreated or stimulated with IFN-γ (500 U/ml) (γDCs) or vit D_3_ (10 ng/ml) (D_3_DCs) or the combination of both (γ/D_3_DCs). Mature DCs, activated with LPS (20 ng/ml) and IFN-γ (500 U/ml) were used as controls where needed. We first evaluated the effect of different treatments on cell viability (Figure 1A). In general, none of the agents or their combinations caused extensive cell apoptosis within 72 h (*n* = 3). Most apoptotic and dead cells were seen in cultures treated with vit D_3_, however this increase was not significant in comparison to mature DCs. The endocytotic capacity of variously treated DCs was compared to immature DCs (Figure 2B, *n* = 4). While the endocytosis of FITC-labeled dextran by D_3_DCs was comparable to iDCs, the endocytotic capacity of γ/D_3_DCs was reduced by 2–3-fold, being similar to that of γDCs. We wanted to determine the effects of combined IFN-γ/vit D_3_ signaling on general DC phenotype (Figure 1C, *n* = 4). While the expression of major DC differentiation markers CD1a and DC-SIGN remained moderate to high irrespective of treatment, the expression of CD14 was strongly induced by vit D_3_, alone or in combination with IFN-γ. Although treatment with vit D_3_ caused down-regulation of DC-SIGN expression, this was not the case with γ/D_3_DCs. The morphology of DCs was assessed using inverted light microscopy (Figure 1D, *n* = 3). All treated cells, namely γDCs, D_3_DCs and γ/D_3_DCs possessed an immature-like appearance, showing absence of dendrite formation, cell clustering and cellular shape similar to iDCs.

### Synergy of IFN-γ and vit D_3_ in Induction of Tolerogenic DC Phenotype

After differentiation from monocytes, DCs were either left untreated or stimulated with IFN-γ, vit D_3_ or IFN-γ and vit D_3_. After 24 h of stimulation, the cells were washed extensively, re-plated and re-stimulated with multimeric CD40 ligand (4 μg/ml) for an additional 24 h. Afterwards, the cell supernatants were collected and analyzed for the presence of IL-1β, IL-6, IL-8, IL-10, IL-12p70, and TNF-α (Figure 2A, *n* = 4). Interestingly, the production of IL-1β, IL-6, IL-8, as well as TNF-α was not suppressed in D_3_DCs and γ/D_3_DCs. Moreover, γ/D_3_DCs showed a significantly induced production of TNF-α in comparison to D_3_DCs and γDCs. On the other hand, the production of IL-12p70 was almost completely inhibited in D_3_DCs and γ/D_3_DCs. In comparison to iDCs and γDCs, the production of IL-10 was much greater in cultures of D_3_DCs and γ/D_3_DCs, with γ/D_3_DCs capable of producing greater quantities of IL-10 than D_3_DCs and with increased IL-10/IL-12p70 ratio (Figure 2B). To analyze the effect of IFN-γ and vit D_3_ on induction of DC tolerogenic phenotype, we measured the expression of inhibitory molecules ILT-3 and PDL-1 on DCs in the presence of different vit D_3_ concentrations, namely 0.1, 1.0, and 10.0 ng/ml, alone or with addition of IFN-γ (Figure 2C, *n* = 5). This titration with vit D_3_ revealed a dose-dependent rise in expression of both inhibitory molecules, with significantly greater expression in the presence of IFN-γ. To test if high expression of ILT-3 and PDL-1 on tolerogenic DCs is sensitive to subsequent activation, we stimulated D_3_DCs and γ/D_3_DCs with mCD40L or LPS for an additional 24 h. Afterwards, the levels of ILT-3 and PDL-1 were determined in the same manner as before. DC activation positively influenced inhibitory molecule expression and was particularly evident when cells were stimulated using LPS (Figure 2D). As shown in Figure 2E (*n* = 5), the expression of ILT-3 and PDL-1 was increased by more than 2-fold on γ/D_3_DCs, compared to either D_3_DCs or γDCs.

### γ/D_3_DCs Display Low Stimulatory Potential

**Figure 3:**
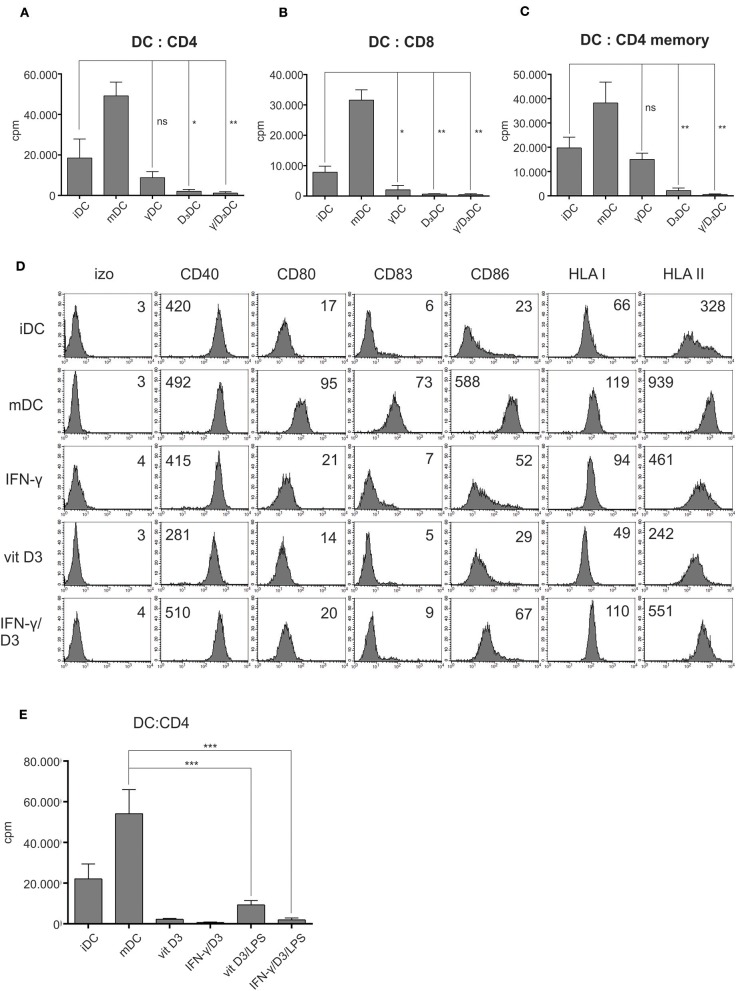
γ/D_3_DCs possess low allo-stimulatory and co-stimulatory potential. Dendritic cells were prepared as described in section Materials and Methods. and used as stimulators in an allogeneic co-culture with **(A)** whole CD4^+^ T cells, **(B)** whole CD8^+^ T cells, or **(C)** memory CD4^+^ T cells. The co-cultures were prepared in 96-well flat bottom plates. After 4 days, the wells were pulsed with tritium-labeled thymidine and proliferation was measured after 18–22 h by liquid scintillation counting. The results are expressed as mean ± SD of counts per minute (cpm) of four independent experiments. **(D)** Variously treated DCs were stained for surface expression of CD40, CD80, CD83, CD86, HLA class I, and HLA class II molecules. Numbers on histograms represent MFI values. Shown is one representative out of three independent experiments performed. **(E)** Dendritic cells were treated with vit D_3_ or vit D_3_ + IFN-γ, followed by 24 h activation using LPS or left inactivated. Subsequently they were used as stimulators in an allogeneic co-culture with CD4^+^ T cells. Proliferation was measured by liquid scintillation counting on day 5, as described above. Immature DCs and DCs matured with LPS + IFN-γ were used as controls. Statistical analysis between individual pairs was performed using Student's unpaired *t*-test (ns, non-significant; ^*^p < 0.05; ^**^p < 0.01; ^***^p < 0.001).

In the next step, we analyzed the basic functional capacity of variously treated DCs. After differentiation from monocytes, as described in section Materials and Methods., the DCs were used as stimulators in an allogeneic co-cultures with either whole CD4^+^ T cells (Figure 3A, *n* = 4), whole CD8^+^ T cells (Figure 3B, *n* = 4), or CD4^+^CD45RO^+^ memory T cells (Figure 3C, *n* = 4). On day 5, the proliferation of T cells in all co-cultures was measured by liquid scintillation counting. In general, γDCs, D_3_DCs, and γ/D_3_DCs all exhibited the capacity to suppress cell proliferation of CD4^+^, CD8^+^, or CD4^+^ memory T cells in comparison to iDCs. While both D_3_DCs and γ/D_3_DCs showed extensive suppressive capacity, γ/D_3_DCs were evidently unique in this regard, which can be observed in co-cultures with CD4^+^ memory T cells (Figure 3C). Since IFN-γ has been reported to induce DC maturation under specific conditions, we found it necessary to analyze the expression of major DC maturation markers after various treatments. In comparison to mDCs, neither γDCs, D_3_DCs, or γ/D_3_DCs showed any noticeable up-regulation of co-stimulatory molecules CD80 and CD86 or the maturation marker CD83 (Figure 3D, *n* = 3). Interestingly, the expression of CD40 molecule was down-regulated only in case of D_3_DCs, but was found similarly or even slightly more expressed on γ/D_3_DCs in comparison to mDCs. To determine whether the low allo-stimulatory potential of γ/D_3_DCs is still retained after exposure to immunogenic signals, we performed an allogeneic co-culture where D_3_DCs or γ/D_3_DCs were used as stimulators after 24 h exposure to LPS (Figure 3E). Activation with LPS caused only a minor increase in T cell proliferation, significantly lower than that of mDCs.

### γ/D_3_DCs Exhibit Functional Tolerogenicity

**Figure 4:**
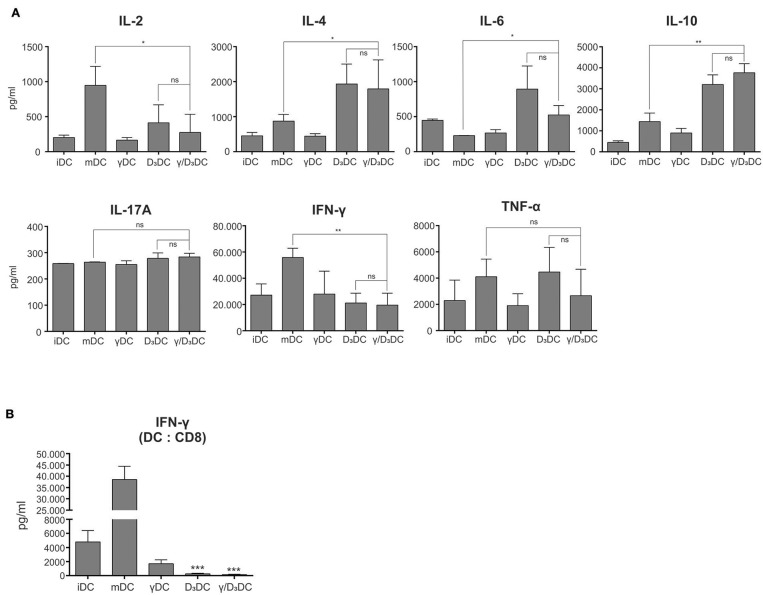
γ/D_3_DCs exhibit functional tolerogenicity. **(A)** The T cell-polarizing capacity of DCs was determined by co-culturing variously-treated DCs with allogeneic naive CD4^+^CD45RA^+^ T cells, as described in section Materials and Methods. After 7–9 days, the T cells were harvested from the culture, extensively washed and re-stimulated using anti-CD2/CD3/CD28 macrobeads for 24 h. At the end of stimulation the supernatants of T cells were analyzed for the presence of IL-2, IL-4, IL-6, IL-10, IL-17A, IFN-γ, and TNF-α. The results are expressed as mean ± SD of four independent experiments. **(B)** The DCs were stimulated with IFN-γ and vit D_3_, alone or in combination and used as stimulators in allogeneic co-cultures with whole CD8^+^ T cells. After 5 days, the supernatants of co-cultures were directly analyzed for the presence of IFN-γ. Results are expressed as mean ± SD of four independent experiments. Statistical significance between individual pairs was calculated using Student's unpaired *t*-test (ns, non-significant; ^*^p < 0.05; ^**^p < 0.01; ^***^p < 0.001).

Dendritic cells treated with IFN-γ, vit D_3_, or IFN-γ + vit D_3_ were assessed for their capacity to induce CD4^+^ T cell polarization and CD8^+^ T cell activation. Either iDCs, mDCs, γDCs, D_3_DCs, or γ/D_3_DCs were used as stimulators in an allogeneic co-culture with naive CD4^+^CD45RA^+^ T cells. After 7–10 day co-culture in RPMI supplemented with 10% human AB serum, the T cells were collected and thoroughly washed. They were then re-plated and re-stimulated using anti-CD2/CD3/CD28 macrobeads for 24 h. In the next step the supernatants were collected and analyzed for the presence of Th1/Th2/Th17 cytokines (Figure 4A, *n* = 4). Cultures with mDCs as stimulators resulted in T cells producing high IL-2 and IFN-γ levels, characteristic of a Th1 response, as expected. The production of IFN-γ by T cells stimulated with D_3_DCs and γ/D_3_DCs was significantly reduced. At the same time, production of IL-10 by T cells stimulated with D_3_DCs and γ/D_3_DCs was significantly increased after T cell receptor stimulation. Interestingly, the production of IL-4 was also increased in this case. Moreover, the levels of TNF-α were substantial in D_3_DCs and γ/D_3_DCs cultures.

Variously treated DCs were also assessed for their capacity to induce IFN-γ production in co-cultures with whole CD8^+^ T cells (Figure 4B, *n* = 4). The levels of IFN-γ were measured directly from the co-cultures after 5 days. While mDCs induced extensive IFN-γ production, this was completely abolished where D_3_DCs or γ/D_3_DCs were used as stimulators. Compared to iDCs, this was ~18- and 33-fold lower for D_3_DCs and γ/D_3_DCs, respectively.

### Tolerogenic Synergy Between IFN-γ and vit D_3_ Is Independent of IDO and Increased 25(OH)D to 1,25(OH)_2_D Conversion

**Figure 5:**
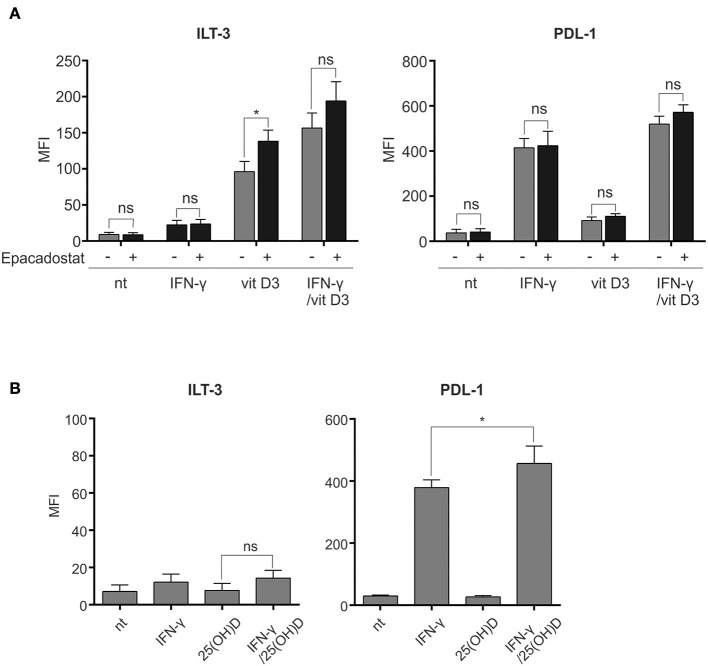
Tolerogenic synergy between IFN-γ and vit D_3_ is independent of IDO activity and increased 25(OH)D-to-vit D_3_ synthesis. **(A)** To exclude the enzymatic activity of IDO for the tolerogenic effects of IFN-γ and vit D_3_, we pre-treated the DCs with a highly specific IDO inhibitor Epacadostat for 2 h. Afterwards, the DCs were stimulated for 24 h and stained for surface expression of ILT-3 and PDL-1, as depicted in the figure. The results are expressed as mean ± SD of MFI from three independent experiments. **(B)** Dendritic cells were cultured in the presence of IFN-γ and 25(OH)D for 48 h, as depicted in the figure. Surface expression of ILT-3 and PDL-1 was evaluated after 48 h. Shown is mean ± SD of four independent experiments. Statistical analysis was made comparing individual pairs and using Student's unpaired *t*-test (ns, non-significant; ^*^p < 0.05).

Both IFN-γ and vit D_3_ have been shown to induce IDO-competence in DCs (12, 22). We set out to study the potential involvement of increased IDO activity for induction of ILT-3^high^PDL-1^high^ γ/D_3_DC phenotype. Dendritic cells differentiated from monocytes were either left untreated or treated with IFN-γ, vit D_3_ or IFN-γ + vit D_3_, as described in section Materials and Methods. Inhibition of IDO was achieved by 2 h -pretreatment of DCs with Epacadostat, a potent IDO inhibitor (23), as indicated in Figure 5A (*n* = 3). After 24 h, variously-treated DCs were analyzed for surface expression of ILT-3 and PDL-1. Pre-treatment with Epacadostat failed to prevent inhibitory molecule up-regulation by IFN-γ and vit D_3_. Interestingly, in case of vit D_3_ treatment, the expression of ILT-3 was further induced with IDO inhibition.

We further wanted to study the contribution of increased 1,25-dihidroxycholecalciferol (vit D_3_) synthesis from 25-hidroxycholecalciferol (25(OH)D) for the observed tolerogenic synergy between IFN-γ and vit D_3_. The rationale for this is the fact that IFN-γ can cause an increase in the above-mentioned metabolic effect by up-regulating the transcription of the responsible enzyme, namely the 1-α-hydroxylase (24, 25). For this purpose, the DCs were either left untreated or treated with IFN-γ, 25(OH)D or IFN-γ + 25(OH)D for 48 h. Afterwards, the surface expression of ILT-3 and PDL-1 was measured (Figure 5B, *n* = 4). We did not observe the expected increase in ILT-3 or PDL-1 upregulation after IFN-γ + 25(OH)D treatment in comparison to IFN-γ treatment alone. However, a slight, although significant, up-regulation was observed in case of PDL-1.

### The Tolerogenic Effect of IFN-γ and vit D_3_ Is Independent of STAT-1 and Can Be Reversed by MEK/ERK and PI3K/Akt/mTOR Pathway Inhibition

**Figure 6:**
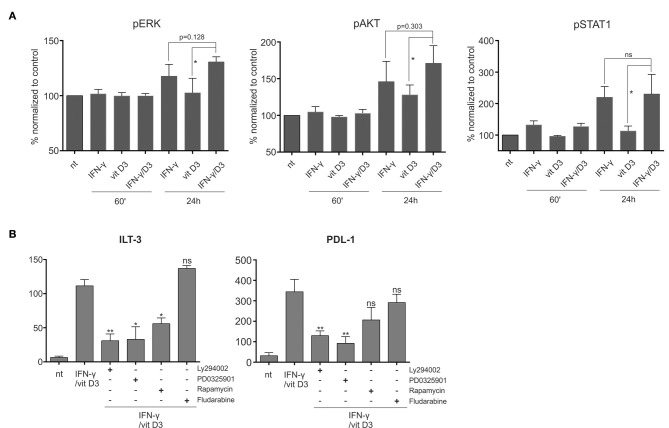
The tolerogenic synergy between IFN-γ and vit D_3_ is dependent on MEK/ERK and PI3K/mTOR signaling. **(A)** The phosphorylation status of ERK, AKT, and STAT-1 signaling proteins was determined. The DCs were stimulated with IFN-γ, vit D_3_, or IFN-γ + vit D_3_. After 1 or 24 h, the phosphorylation state of intra cellular proteins was frozen by direct addition of PFA into culture medium. The cells were then permeabilized using ice-cold methanol and stained intracellulary against pERK, pAkt, and pSTAT-1. Results are expressed as % of MFI normalized against untreated controls. Shown is mean ± SD of five independent experiments. **(B)** Dendritic cells were pre-treated with either inhibitor of PI3K (Ly294002), MEK/ERK (PD0325901), mTOR (rapamycin), or STAT-1 (fludarabine) for 2 h. Afterwards, the DCs were stimulated using IFN-γ and vit D_3_, for 24 h. After stimulation, the surface expression of ILT-3 and PDL-1 was analyzed. Results are expressed as mean ± SD of four independent experiments. Statistical analysis was performed using Student's unpaired *t*-test for determining significance between individual pairs (ns, non-significant; ^*^p < 0.05; ^**^p < 0.01).

We evaluated the activity of signaling pathways associated with both IFN-γ and vit D_3_ signaling, namely the MEK/ERK, PI3K/Akt/Mtor, and STAT-1 pathway. To determine the activation of various signaling pathways after various treatments, we measured the intracellular levels of phosphorylated ERK, Akt, and STAT-1 proteins. Fully differentiated DCs were either untreated or treated with IFN-γ, vit D3, or IFN-γ + vit D_3_. After 60 min or 24 h, PFA was added directly into culture medium to assure“freezing” of the phosphorylated states. After permeabilization, the DCs were stained with corresponding mAbs against pERK, pAkt or pSTAT-1 (Figure 6A, *n* = 5). Both IFN-γ and vit D_3_ increased the phosphorylation state of ERK and Akt proteins, particularly after 24 h. We observed a further increase in ERK and Akt activation when DCs were simultaneously stimulated with IFN-γ and vit D_3_. Although the significance of this increase could only be determined in comparison to vit D_3_ stimulation, there was a clear trend for higher phosphorylation levels in γ/D_3_DCs. The phosphorylation of STAT-1 was low in vit D_3_-stimulated cells, however, the presence of vit D_3_ did not lower pSTAT-1 levels in γ/D_3_DCs.

Next, we wanted to determine how inhibition of a particular signaling pathway influences the tolerogenic phenotype of γ/D_3_DCs. For this purpose we pre-treated the DCs for 2 h with inhibitors of PI3K (Ly294002), MEK (PD0325901), mTOR (Rapamycin), and STAT-1 (Fludarabine). Afterwards, the cells were stimulated as depicted in Figure 6B (*n* = 4). While inhibition of STAT-1 had no effect on expression of either ILT-3 or PDL-1, there was a marked reduction in expression of both inhibitory molecules particularly in the presence of PI3K and MEK inhibitor. Although the inhibition of mTOR also resulted in decreased expression of ILT-3 and PDL-1, this was seen to a lesser extent and could not be found significant in case of PDL-1.

### The Function of γ/D_3_DCs Is Dependent on PDL-1 and Their Active Suppression Exerted Primarily Toward CD8^+^ T Cells

**Figure 7:**
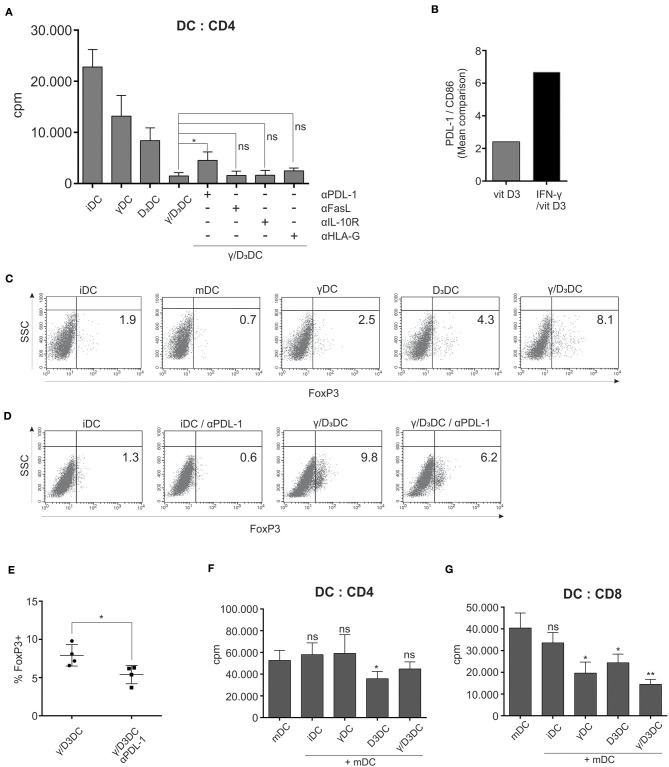
PDL-1 is important for γ/D_3_DC's function and their active immunosuppression is exerted primarily toward CD8^+^ T cells. **(A)** Differentially treated DCs were used as stimulators in an allogeneic mixed lymphocyte reaction. In parallel co-cultures, blocking mAbs were used against PDL-1, IL-10R, FasL, and HLA-G. After 5 days, cell proliferation was measured by liquid scintillation counting. Results are expressed as mean ± SD of determined counts per minute of four independent experiments. **(B)** The ratio of PDL-1 vs. CD86 expression is depicted for D_3_DCs and γ/D_3_DCs. The ratio was determined based on mean MFI values of analyzed markers. **(C)** The capacity of variously-treated DCs for induction of FoxP3 expression from naive T cells was determined. Dendritic cells were used as stimulators of allogeneic naive CD4^+^CD45RA^+^ T cells. After 7 days, the T cells were collected, fixed, permeabilized, and stained intracellularly for FoxP3 expression. Shown is one representative out of four independent experiments performed. The numbers in quadrants represent the percentage of positive cells. **(D)** Parralel co-cultures were performed, where naive CD4^+^CD45RA^+^ T cells were stimulated with immature DCs or γ/D_3_DCs in the presence or absence of blocking anti-PDL-1 mAb. At the end of co-culture, the percentage of FoxP3-expressing T cells was determined. **(E)** Shown is mean ± SD of percentage of FoxP3^+^ T cells from co-cultures where γ/D_3_DCs were used as stimulators with or without PDL-1-blocking mAb. **(F)** Variously treated DCs were added to co-cultures of mDCs and allogeneic CD4^+^ T cells or **(G)** allogeneic CD8^+^ T cells. On day 4 the cultures were pulsed with tritium-labeled thymidine and proliferation was measured after 18–22 h by liquid scintillation counting. Shown is mean ± SD of four independent experiments. Statistical analysis was performed between individual pairs using Student's unpaired t-test (ns, non-significant; ^*^p < 0.05; ^**^p < 0.01).

To gain further insight into molecular mechanisms underlying the extensive tolerogenicity of γ/D_3_DCs, we performed additional functional assays in the presence of various blocking Abs. Variously stimulated DCs, prepared as described in section Materials and Methods., were used as stimulators in a mixed lymphocyte reaction with responding whole CD4^+^ T cells (Figure 7A, *n* = 4). In parallel, co-cultures were treated with blocking Abs against PDL-1, IL-10R, FasL, and HLA-G. As expected, γ/D_3_DCs caused a substantial suppression of T cell proliferation. In the presence of blocking Abs, namely anti-IL-10R, anti-FasL, and anti-HLA-G, we did not observe significant reversion of T cell proliferation. On the other hand, administration of anti-PDL-1 successfully reinvigorated the stimulatory capacity of γ/D_3_DCs to a certain extent. By looking at the ratio of PDL-1 vs. CD86 expression on DCs, which is an important characteristics of DC tolerogenicity, we observed a substantially higher values on γ/D_3_DCs compared to D_3_DCs (Figure 7B).

We assessed the capacity of variously treated DCs to induce Foxp3 expression from naive CD4^+^CD45RA^+^ T cells. In a 7 day co-culture, γ/D_3_DCs showed a superior capacity to induce or preserve CD4^+^FoxP3^+^ T cell populations. On average, co-cultures with γ/D_3_DCs displayed a 2-fold greater percentage of FoxP3^+^ T cells compared to D_3_DCs, 3-fold greater than γDCs and ~4-fold greater than iDCs (Figure 7C, *n* = 4). Due to functional importance of PDL-1 in induction of T cell proliferation, we performed parallel experiments where blocking anti-PDL-1 mAb was added to co-cultures of naive T cells stimulated with iDCs or γ/D_3_DCs (Figure 7D, *n* = 4). The neutralization of PDL-1 resulted in downregulation of FoxP3-positive cells by ~40% (Figure 7E). However, when these FoxP3^+^ T cells were tested in a Treg suppression assay we could not confirm their suppressive activity (data not shown).

We therefore further investigated the active suppression of DCs in a DC suppression assay, to test how variously treated DCs can inhibit T cell proliferation stimulated by mDCs. Interestingly, γ/D_3_DCs were not very efficient at suppressing mDC-induced CD4^+^ T cell proliferation, which was better achieved using tolerogenic DCs induced by vit D_3_ alone (Figure 7F). Due to great inhibitory potential of γ/D_3_DCs toward CD8^+^ T cells, the DC suppression assay was also studied in this context. Contrary to co-cultures with CD4^+^ T cells, γ/D_3_DCs significantly inhibited the proliferation of CD8^+^ T cells induced by mDCs and were ~2-fold more efficient than D_3_DCs (Figure 7G).

## Discussion

In this paper we present evidence which highlights the importance of concomitant signaling of two important endogenous immunomodulators, namely IFN-γ and vit D_3_, in induction of a potent and unique DC tolerogenic state. Dendritic cells possess extensive plasticity in acquiring numerous “flavors” of their activation state, being either immunogenic or tolerogenic (26). In many cases, their acquisition of a particular activation state is known to benefit from activation of multiple, frequently overlapping signaling pathways. A typical example can be drawn from rich experiences in achieving type 1 DC polarization, which has been extensively studied due to its importance in anti-cancer DC activation protocols. For instance, concomitant signaling via various toll-like receptors (TLRs) activation and by various inflammatory cytokines (e.g., type I or II IFNs) is known to greatly induce the DC's co-stimulatory potential and the capacity to induce strong Th1 responses (27–29).

Vitamin D_3_ exerts pleiotropic immunomodulatory effects in context of immune cell type specificity. While it is generally known as a potent inducer of tolerogenic DCs, it can augment activation of inflammatory pathways in monocytes (6). It is in the monocyte/macrophage lineage where the cooperation between vit D_3_ and IFN-γ has first been demonstrated (14) and since then, discoveries in mechanisms underlying the cellular actions of both agents have suggested possible interactions in other immune cell types, such as DCs. We first made an assessment of general effects caused by concomitant IFN-γ/vit D_3_ treatment of fully differentiated, monocyte-derived DCs. Since vit D_3_ has been shown to affect DC viability (30), we analyzed the percentage of cell death in variously treated DCs (Figure 1A). Indeed, cultures with D_3_DCs showed slightly increased apoptosis after 72 h, although not in a significant manner compared to either iDCs or mDCs. The survival of γ/D_3_DCs was generally greater. The maturity of their activation state was in part determined by analysis of their endocytotic capacity, which was lower for both γDCs and γ/D_3_DCs (Figure 1B), however still significantly higher compared to mDCs. This was reflected in terms of both phenotype and morphology (Figures 1C,D), with γ/D_3_DCs displaying typical immature morphology with no signs of dendrite formation or cell clustering. Phenotypically, treatment with IFN-γ and vit D_3_ did not cause particular down-regulation of major DC differentiation markers such as CD1a or DC-SIGN. However, it did result in reappearance of CD14 expression, both with D_3_DCs and γ/D_3_DCs (Figure 1C). There are not many studies that analyzed the effect of vit D_3_ on already established, monocyte-derived DCs. Penna et al. did study its effects on native myeloid and plasmacytoid DCs, but without detailed analysis of differentiation markers (31). However, it is well-known that vit D_3_ strongly deviates monocyte-to-DC differentiation toward tolerogenic APCs, while preserving the high expression of CD14 (30).

From the functional standpoint, we first wanted to evaluate the response of γ/D_3_DCs to potential T cell-derived signaling by stimulating variously treated DCs with CD40L and observing their cytokine profile. In contrast to γDCs, where treatment with IFN-γ resulted in a prominent priming response for extensive IL-12p70 production (32), there was a strong downregulation of IL-12p70 production in γ/D_3_DCs (Figure 2A). In contrast, IL-10 production was strongly up-regulated. Interestingly, the production of IL-10 was higher in γ/D_3_DCs in comparison to D_3_DCs, which consequently led to a more than 2-fold greater IL-10/IL-12p70 ratio (Figure 2B). Vitamin D_3_ itself has been demonstrated to act as a strong inducer of IL-10 expression in DCs (33). One of first studies researching the possible interactions between STAT-1 and VDR was performed by Vidal et al., who showed that mutual stimulation of macrophages with IFN-γ and vit D_3_ results in physical interactions between STAT-1 and VDR. The interaction between both proteins resulted in decreased binding of VDR to vitamin D responsive elements (VDREs) on DNA (17) which resulted in decreased expression of 24-hydroxylase, a key enzyme in vit D_3_ inactivation. Our results suggest that a potential STAT-1-mediated antagonism in relation to VDR signaling is irrelevant in context of IL-10 transcription. We also noticed a differential production in other cytokines. In particular, the levels of TNF-α were greatly induced in γ/D_3_DCs culture supernatants (Figure 2A) in comparison to D_3_DCs. Similarly, there was an increase in production of IL-1β and IL-6.

The tolerogenic status of DCs treated with vit D_3_ is strongly associated with expression of specific inhibitory molecules, such as ILT-3 and PDL-1 (31, 34). We performed a titration if vit D_3_ in the presence of IFN-γ to analyze potential synergy on this tolerogenic signature (Figure 2C). The presence of IFN-γ greatly induced the expression of both ILT-3 and PDL-1, in comparison to treatment with vit D_3_ alone and this expression was further induced by exposure to immunogenic signals such as LPS or CD40L (Figure 2D). This synergy was clearly evident in expression of ILT-3, since IFN-γ alone does not act as a strong inducer of ILT-3 on its own (Figure 2E). Although the exact ligand/receptor pathway of ILT-3 is unknown as of yet, its expression is strongly correlated with DC tolerogenicity and the presence of both soluble and membrane-bound ILT-3 have been associated with increased generation of T suppressor cells (35). The tolerogenicity of γ/D_3_DCs was confirmed in an allo-stimulatory lymphocyte reaction. Dendritic cells treated simultaneously with IFN-γ and vit D_3_ displayed extensive suppression of whole CD4^+^ T cells (Figure 3A). This was also the case when either CD8^+^ T cells or CD4^+^ memory T cells were used as responders (Figures 3B,C, respectively). γ/D_3_DCs were most capable of inhibiting responses of all tested T cell populations, which is clearly seen in case of CD4^+^ memory T cells when compared to D_3_DCs (Figure 3C). To gain further insight into γ/D_3_DCs' co-stimulatory capacity we performed a broad phenotypical analysis of DC maturation markers. Previously, Frasca et al. reported on an alleged capacity of IFN-γ to induce maturation of DCs (36). In addition, vit D_3_ treatment can support NF-κB and MAPK activation in human monocytes (6). Stimulation of DCs with IFN-γ and vit D_3_ did cause a slight increase in CD40, CD86, as well as the expression MHC class II molecules (Figure 3D). However, we did not observe any up-regulation of CD80 or the maturation marker CD83. In this context, there is an extensive dominance regarding the induction of inhibitory vs. co-stimulatory molecules. After prolonged exposure to LPS, the low allo-stimulatory function was preserved in D_3_DCs and even to a further extent in γ/D_3_DCs (Figure 3E), suggesting resistance to maturation. When acting by itself, IFN-γ is a strong priming agent for type 1 DC polarization, a characteristic which seems lost in associated signaling with vit D_3_ pathway and further supported by the incapacity of γ/D_3_DCs to produce IL-12p70 upon CD40L stimulation (Figure 2A).

Dendritic cell tolerogenicity is frequently associated with increased capacity of Treg induction and/or suppression of effector T cell responses. After single stimulation, γ/D_3_DCs induced T cell populations from naive CD4^+^CD45RA^+^ T cells, capable of producing increased IL-10 levels upon activation. However, we could not determine a significant increase in this capacity compared to D_3_DCs (Figure 4A). The T cells from γ/D_3_DC co-cultures also produced significantly lower levels of IL-2 and IFN-γ compared to co-cultures where mDCs were used as stimulators. Type 1 regulatory T cells (Tr1) are characterized by high IL-10 production and low production of IL-4. The still present production of IL-4 by activated T cells in our case is normal, since induction of fully differentiated Tr1 cells usually requires multiple rounds of stimulation, before T cells loose the capacity to produce IL-4 (37, 38). Furthermore, γ/D_3_DCs also contributed to extensive suppression of CD8^+^ T cell responses. This was observed after complete absence of IFN-γ production in co-cultures with whole CD8^+^ T cells (Figure 4B).

Interferon-γ and vit D_3_ can both induce IDO-competence in human DCs (22, 39). Increased IDO activity in DCs causes environmental tryptophan starvation which leads to suppression of effector T cell responses. In addition, tryptophan degradation also induces tolerogenic phenotype in DCs, up-regulating inhibitory molecules such as ILT-3 (40). We tested whether potential increase in IDO-competence caused by IFN-γ and vit D_3_ underlies the extensive expression of inhibitory molecules on γ/D_3_DCs. For this purpose we pre-treated the cells with Epacadostat, a highly effective and specific IDO inhibitor (23). Blockade of IDO activity prior stimulation did not negatively affect the expression of either ILT-3 or PDL-1 on DCs (Figure 5A). Moreover, we even noticed a marginal increase in ILT-3 expression in Epacadostat-treated cultures. An important modification of vitamin D into its active form is the 1α-hydroxylation of 25(OH)D to 1,25(OH)_2_D or vit D_3_ by the enzyme CYP27B1 (41). In macrophages, IFN-γ has been shown to induce the expression of CYP27B1. In this manner, we wanted to assess to what extent IFN-γ-induced synthesis of vit D_3_ from 25(OH)D could contribute to the tolerogenic effect observed by both agents. We cultured DCs in the presence of IFN-γ and 50 ng/ml of 25(OH)D to observe any potential induction of tolerogenic phenotype. Treatment of DCs with 25(OH)D alone did not cause any changes in expression of ILT-3 or PDL-1 (Figure 5B). When treated in the presence of IFN-γ, the increase of inhibitory molecules was marginal at best. We did not notice any significant increase in expression of ILT-3, whereas the expression of PDL-1 was increased by ~15% (*n* = 4). The main tolerogenic synergy between vit D_3_ and IFN-γ in DCs therefore does not stem from increased synthesis of vit D_3_, but is more likely the result of direct interaction between both signaling pathways.

More recently, Ferreira et al. demonstrated that the tolerogenic effect of vit D_3_ in DCs is directly associated with the activation of PI3K/Akt/mTOR signaling pathway (42). On the other hand, the well-known pleiotropism of IFN-γ is also reflected by its capacity of STAT-1-independent signaling. In this manner, IFN-γ signaling is known to activate both PI3K/Akt, as well as ERK pathways (43). The Ras-ERK and PI3K/Akt/mTOR signaling pathways are the chief mechanisms for controlling cell survival, differentiation, and metabolism in response to external cues. In addition to their independent signaling both pathways have been shown to interact extensively and exert bidirectional regulation in both positive and negative manner (44). For this reason we analyzed the phosphorylation levels of ERK, Akt, and STAT-1 in variously treated DCs (Figure 6A). There was a positive trend for activation of both ERK and Akt in DCs simultaneously stimulated with IFN-γ and vit D_3_ in comparison to single agent treatments. Interestingly, the phosphorylation level of STAT-1 was not reduced by vit D_3_ treatment. To further assess the contribution of these signaling pathways for DC's tolerogenic phenotype, we used specific inhibitors of both pathways, as well as inhibitors of STAT-1. The inhibition of STAT-1 did not cause significant changes in the capacity of IFN-γ and vit D_3_ to induce inhibitory molecule expression. On the contrary, the inhibition of PI3K (by Ly294002) and MEK (by PD0325901) greatly inhibited the expression of both ILT-3 and PDL-1. While inhibition of mTOR also showed a similar effect, it was not as extensive (Figure 6B).

We finally analyzed the contribution of various tolerogenic elements for γ/D_3_DCs function. We used anti-PDL-1, anti-HLA-G, anti-FasL, and anti-IL10R neutralizing mAbs in a mixed lymphocyte reaction with γ/D_3_DCs (Figure 7A). As depicted, inhibition of PDL-1, but not others, could partially restore the low allo-stimulatory capacity of γ/D_3_DCs, suggesting the importance of active induction of immunosuppression by PDL-1 as opposed to general lack of co-stimulatory capacity. As shown in Figure 7B, the PDL-1/CD86 ratio on γ/D_3_DCs is ~3-fold greater compared to that observed on D_3_DCs. γ/D_3_DCs also possess extensive capacity to increase the percentage of FoxP3^+^ T cells during co-cultures with naive T cells (Figure 7C). The percentage of FoxP3^+^ after 7 days of co-culture with CD4^+^CD45RA^+^ T cells was 2-fold greater (*n* = 4) in comparison to co-cultures where D_3_DCs were used as stimulators. The neutralization of PDL-1 lowered the percentage of FoxP3-expressing T cells by almost 40% (*n* = 4) (Figures 7D,E). Our results are in concordance with those obtained by Unger et al. who demonstrated the essential role of PDL-1 in Treg induction by vit D_3_-induced tolerogenic DCs (45). However, in our subsequent analysis we could not observe functional suppression of T cells from co-cultures with γ/D_3_DCs when performing a Treg suppression assay (data not shown). This led us to speculate that CD4 T cells are not the primary subset sensitive to tolerance induction by γ/D_3_DCs. To confirm this we performed a DC suppression assay, where we added variously treated DCs to allogeneic co-cultures of mDCs with either CD4^+^ or CD8^+^ T cells. Interestingly, while D_3_DCs could inhibit mDC-induced proliferation of CD4^+^ T cells, γ/D_3_DCs were inefficient in this manner (Figure 7F). On the contrary, γ/D_3_DCs were very efficient at suppressing mDC-induced proliferation of CD8 T cells, being twice as effective as D_3_DCs (Figure 7G).

Overall, our study identifies a novel role of double signaling mediated by vit D_3_ and IFN-γ in inducing strong and unique DC tolerogenicity, capable of exerting active tolerance induction particularly in context of CD8^+^ T cells. Since both IFN-γ and vit D_3_ are well-recognized modulators of DC immunity under pathological conditions, their synergistic effects could play an important role in regulating immune responses in various autoimmune and other inflammatory diseases. Furthermore, in the quest for generating novel protocols for tolerogenic APC therapies, our results contribute to understanding of mechanisms underlying the manipulation of DCs toward increased tolerogenic characteristics.

## Data Availability Statement

All datasets generated for this study are included in the article/supplementary material.

## Author Contributions

UŠ designed and performed the research. UŠ and PR wrote the manuscript.

### Conflict of Interest

The authors declare that the research was conducted in the absence of any commercial or financial relationships that could be construed as a potential conflict of interest.
